# Prognostic potential and pathological validation of a diagnostic application using Raman spectroscopy in the characterization of degenerative changes in the cartilage of the humeral head

**DOI:** 10.1117/1.JBO.27.11.115002

**Published:** 2022-11-10

**Authors:** Ryuji Asaoka, Hiroshi Kiyomatsu, Hiromasa Miura, Akihiro Jono, Tomofumi Kinoshita, Masaki Takao, Takashi Katagiri, Yusuke Oshima

**Affiliations:** a University of Toyama, Graduate School of Science and Engineering, Toyama, Japan; b Ehime University, Graduate School of Medicine, Department of Bone and Joint Surgery, Toon, Japan; c Kyushu Rosai Hospital, Kitakyushu, Japan; d University of Toyama, Faculty of Engineering, Toyama, Japan; e University of Toyama, Research Center for Pre-Disease Science, Toyama, Japan; f Oita University, Faculty of Medicine, Yufu, Japan

**Keywords:** Raman spectroscopy, cartilage, osteoarthritis, humeral cartilage, shoulder joint

## Abstract

**Significance:**

Raman spectroscopy is a well-established analytical method in the fields of chemistry, industry, biology, pharmaceutics, and medicine. Previous studies have investigated optical imaging and Raman spectroscopy for osteoarthritis (OA) diagnosis in weight-bearing joints such as hip and knee joints. However, to realize early diagnosis or a curable treatment, it is still challenging to understand the correlations with intrinsic factors or patients’ background.

**Aim:**

To elucidate the correlation between the Raman spectral features and pathological variations of human shoulder joint cartilage.

**Approach:**

Osteoarthritic cartilage specimens excised from the humeral heads of 14 patients who underwent shoulder arthroplasty were assessed by a confocal Raman microscope and histological staining. The Raman spectroscopic dataset of degenerative cartilage was further analyzed by principal component analysis and hierarchical cluster analysis.

**Results:**

Multivariate association of the Raman spectral data generated three major clusters. The first cluster of patients shows a relatively high Raman intensity of collagen. The second cluster displays relatively low Raman intensities of proteoglycans (PGs) and glycosaminoglycans (GAGs), whereas the third cluster shows relatively high Raman intensities of PGs and GAGs. The reduced PGs and GAGs are typical changes in OA cartilage, which have been confirmed by safranin–O staining. In contrast, the increased Raman intensities of collagen, PGs, and GAGs may reflect the instability of the cartilage matrix structure in OA patients.

**Conclusions:**

The results obtained confirm the correlation between the Raman spectral features and pathological variations of human shoulder joint cartilage. Unsupervised machine learning methods successfully yielded a clinically meaningful classification between the shoulder OA patients. This approach not only has potential to confirm severity of cartilage defects but also to determine the origin of an individual’s OA by evaluating the cartilage quality.

## 1 Introduction

Osteoarthritis (OA) is the most common disease in articular cartilage. It is a major cause of joint pain and disability worldwide. In OA, degradation of the cartilage matrix, subchondral bone sclerosis, and inflammation of the synovial membrane cause physiological and anatomical alterations of the joint tissues. As OA progresses, the typical features observed on X-rays are joint space narrowing, morphological changes in the bone, and ectopically formed calcified tissue as OA progresses.^1^^–^^3^ Cartilage matrix degradation is often viewed as irreversible. The standard cure for end-stage OA is limited to pain management with joint replacement. In addition, cartilage is not visualized on X-rays, making early diagnosis difficult, and the onset mechanism of OA is not fully understood.^4^^,^^5^ An improved understanding of the pathogenesis combined with practical methods to characterize the cartilage matrix biochemically may contribute to the prevention and treatment of early-stage OA.

During the last decade, optical imaging and spectroscopic modalities including optical coherent tomography, second harmonic generation microscopy, and Raman spectroscopy have been employed to evaluate degenerative changes in the cartilage matrix of murine models or human specimens.^6^^–^^16^ In particular, Raman spectroscopy is a nondestructive analytical tool, which provides information about the molecular structure, polarization, crystallinity, and conformation. The cartilage matrix mainly consists of Type II collagen and proteoglycans (PGs) underlying a collagen fibril meshwork.^17^ PGs can be subdivided into core proteins and glycosaminoglycans (GAGs) covalently attached to the core proteins. GAGs are composed of repeating units of di-saccharides, and sulfated GAG chains bind to core protein to form PGs. A progressive loss of cartilage components, mainly PGs and GAGs, may be a leading progression of OA.^18^ Raman spectroscopy can characterize disorder of these molecules. In the last two decades, numerous Raman spectroscopic studies have been published for biological and medical applications. Raman spectroscopy is a reliable technique to detect the disease state of cells and tissues in situ. Because its label-free, Raman spectroscopy holds promise for less-invasive diagnoses or optical biopsies in clinical settings.^19^

Previous studies have investigated optical imaging and Raman spectroscopy for OA diagnosis. Examples include our previous work on a drug- and exercise-induced model,^10^^,^^16^ post-traumatic animal models,^12^^,^^13^ and human cartilage tissue in which loading stress may largely affect the knee^11^ or hip joints.^14^^,^^15^ Generally, mechanical stress is a major cause of OA progression in the articular of hind limbs or lower limbs, which are physiologically exposed to loadings (weight-bearing joints) in both human and animal models. To realize early diagnosis or a curable treatment, it is crucial to understand the correlations with intrinsic factors such as sex, age, osteoporosis, hypertension, hyperglucosemia, and other complications.^20^^–^^23^ Thus, using an upper joint such as the shoulder or elbow, which are nonweight bearing joints for Raman spectroscopic analysis of cartilage degeneration may be useful.^24^ However, research about Raman analysis of human humeral cartilage has yet to be published. In this study, we demonstrate the pathological validation of degenerative cartilage of the shoulder joint in patients using Raman microscopy. Raman spectroscopy and subsequent analysis may reveal the relationship between the molecular disorder of the cartilage matrix and clinical symptoms. Eventually, the clinical integration of Raman spectroscopy in arthroscopy may be realized.

## 2 Materials and Methods

### 2.1 Human Cartilage Samples

Surgically resected specimens were provided for this study. This research protocol was approved by the ethics committee of Ehime University Hospital (approval ID #1507017). We measured the cartilage layer of humeral heads taken from patients who underwent artificial shoulder joint replacement surgery (14 patients). The severity of OA was radiographically determined by the Kellgren–Lawrence (K–L) score in the preoperative diagnosis. Intraoperative assessments of degeneration in the humeral cartilage were conducted in accordance with the International Cartilage Repair Society (ICRS) grading system.

The humeral head was fixed with formalin immediately after resectioning. The cartilage tissue around the top of the humeral head was cut out and embedded in the O.C.T compound. Then it was frozen, thinly sliced (25-μm thick), and placed on a silica window (Micro-chamber INT-750, INTROTEC, Kanagawa, Japan) for Raman analysis. The Raman spectrum was obtained by a laser Raman microscopy system. Continuous sections (7  μm) were prepared for histopathological examination via Safranin–O staining (Saf–O–staining) and Hematoxylin and Eosin staining (HE–staining).

### 2.2 Raman Microscopy

We employed a custom-designed confocal Raman microscopy system to acquire Raman spectra of the cartilage tissues. A slice section was placed onto an inverted microscope (Ti-E, Nikon, Tokyo, Japan). An excitation laser (Samba 532 nm 150 mW, Cobolt, Solna, Sweden) was connected via an optical fiber-coupling device (RPM-532, Airix, Tokyo, Japan) to the microscope. The excitation laser was irradiated onto the specimen with ×20 objective lens (CFI Plan Apochromat Lambda D 20X, Nikon, Tokyo, Japan). The backward scattering was collected at the same objective and returned along the same optical path. Raman scattering light was isolated by a dichroic beam splitter (LPD02-532RU-25x36x1.1, Semrock, Northbrook, Illinois) and a long-pass filter (LP03-532RE-25, Semrock). The scattering light was detected via a multimode fiber (core diameter 50  μm), which was connected to a spectrometer (SP2150, Teledyne Princeton Instruments, Trenton, New Jersey) with a CCD (iVac, ANDOR TECHNOLOGIES, Abingdon, UK). The Raman spectra of each specimen were acquired with an exposure time of 60 s and two times accumulation.

### 2.3 Raman Spectral Data Processing

Baseline corrections of the obtained Raman spectral data were performed by quartic polynomial curve fitting with the “polyfit” function of MATLAB (MathWorks, Natick, Massachusetts) prior to quantitative and statistical analyses.

### 2.4 Multivariate Analysis of Raman Spectral Data

For multivariate analysis, a Raman spectral dataset was prepared for principal component analysis (PCA). Raman spectra, which were obtained from multiple acquisitions at different spots in the same specimen, were averaged to create one spectrum to compensate for the site dependency. In the PCA procedure, wavenumbers (${\mathrm{cm}}^{-1}$) of the spectral dataset were regarded as variables. The variables were transformed into principal components (PCs).^25^ Discrimination models were built based on the partially extracted spectral regions for the fingerprint region (770 to $1700\;{\mathrm{cm}}^{-1}$). Hierarchical cluster analysis (HCA) was applied to the specimen datasets of the statistics.^26^ The analysis was performed using MATLAB (MathWorks, Natick, Massachusetts) with the “cluster” function.

### 2.5 Statistical Analysis

Statistical data were presented as the average ± the standard error of the mean. The significance among the clusters of samples was determined using the Steel–Dwass test. The significance between the two groups was determined using the Mann–Whitney U test. These tests were performed using JMP Pro (SAS Institute, Cary, North Carolina). The values of p<0.05 were considered statistically significant.

## 3 Results

**Fig. 1:**
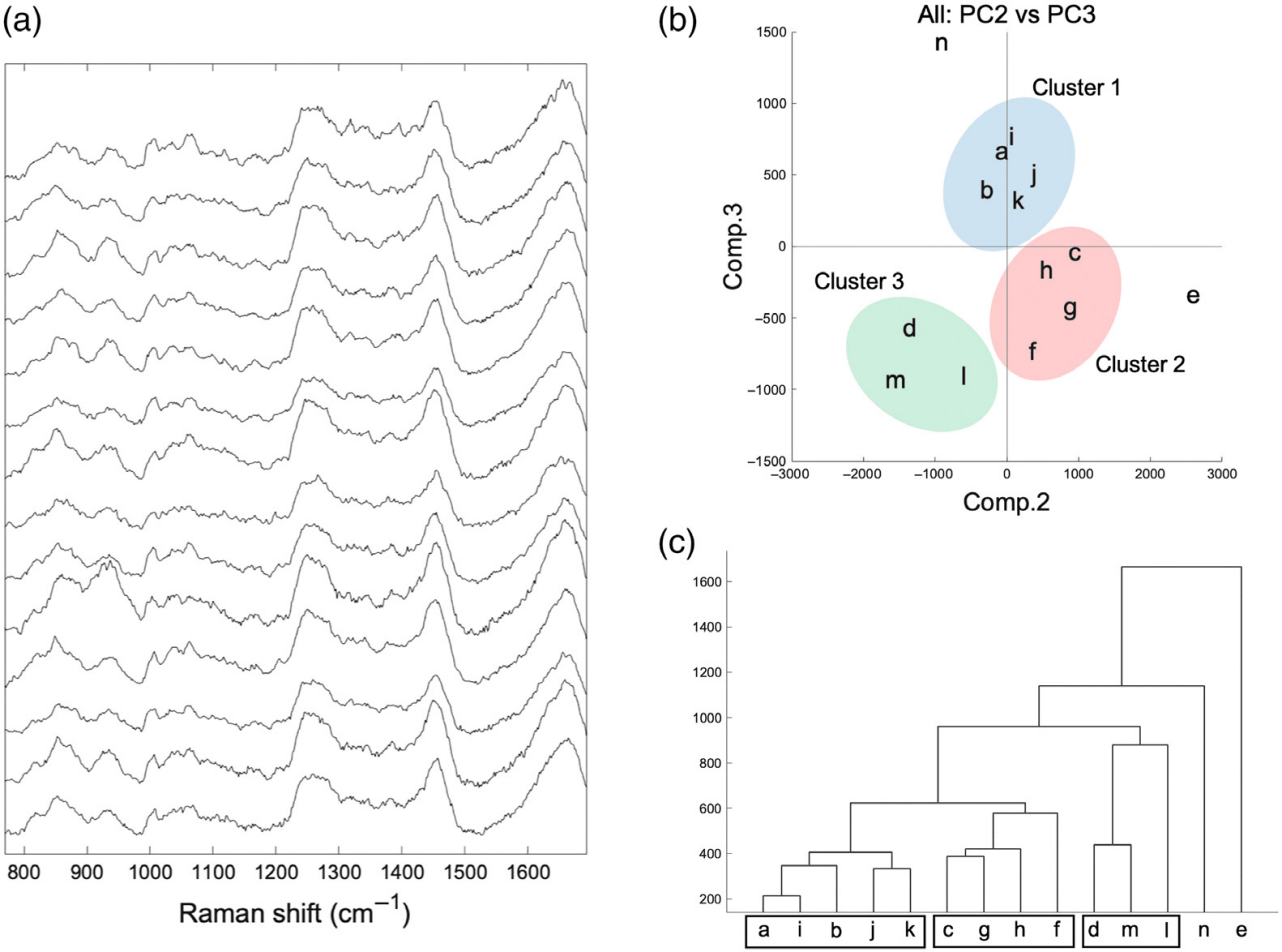
(a) Mean (n=42 spectra) Raman spectra obtained from cartilage tissue of 14 human humeral heads. (b) Multivariate analysis-based PCA and HCA algorithm classifies different characteristic grades of osteoarthritis into separate clusters (Cluster 1: blue, Cluster 2: red, and Cluster 3: green). (c) HCA of the second and third principal components obtained by PCA.

Forty-two Raman spectra were collected (three spectra were acquired for each specimen, n=14) and subjected to multivariate analysis. Figure 1(a) depicts the mean Raman spectra of the cartilage tissue in the humeral heads obtained from 14 patients. Although the spectra showed similarities and the same peak positions, their spectral features differed. PCA was performed to clarify the spectral similarities or differences [Fig. 1(b)]. PC1 was negligible due to the variation in the spectral intensity among the specimens (data not shown). Thus, the mean spectrum from each patient was characteristically separated on the second and third principal components (PC2 and PC3). HCA was also performed using the principal component score (PC score) of PC2 and PC3 [Fig. 1(c)]. Three major clusters (cluster 1, 5 patients; cluster 2, 4 patients; cluster 3, 3 patients) were defined. Two patients (#e and #n) were not included in any cluster [Figs. 1(b) and 1(c)].

**Fig. 2:**
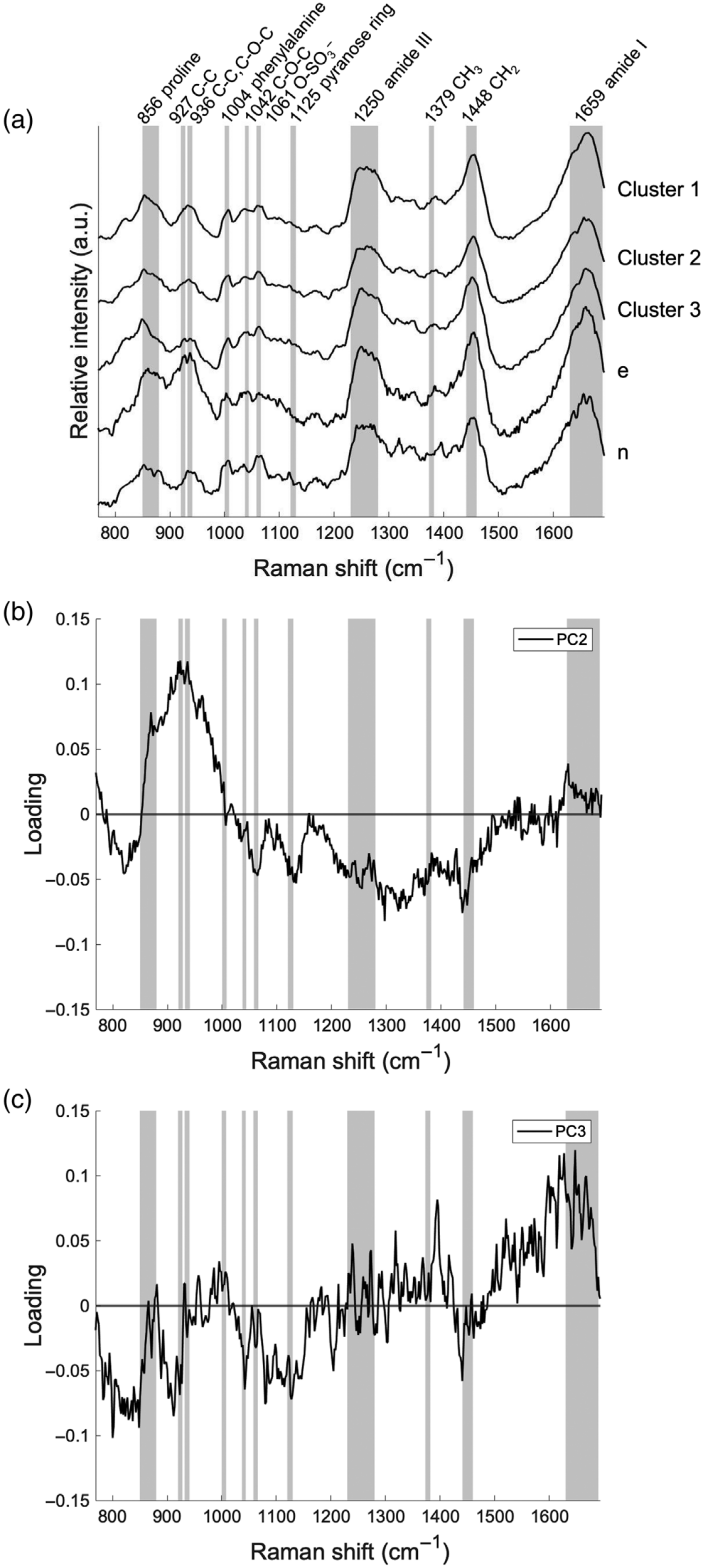
(a) Mean Raman spectra for each cluster and for samples that could not be clustered. (b), (c) Loading plots associated with PC2 and PC3, which are mainly responsible for the discrimination between samples of different clusters of osteoarthritis.

To elucidate what the clustering reflects in the spectral features, which were derived from the molecular composition in the cartilage matrix, the Raman peaks associated with the cartilage matrix were assigned by referring to the literature (Table 1). Figure 2(a) shows the mean spectra of the three clusters, #e, and #n. We then focused on 11 Raman bands, which were mainly assigned to collagen, PGs, and GAGs. The main Raman band assignments for cartilage tissues were consistent with previous reports [Figs. 2(a) and Table 1]. The three clusters showed similar spectral features. However, the spectral features of #e and #n differed significantly from the three clusters.

**Table 1:** Raman peaks assignments of the articular cartilage molecular fingerprint, obtained from OA tissues.

Raman shift (${\mathrm{cm}}^{-1}$)	Assigned bond/molecules	Component
850–880	C─C stretching	Collagen
856–858	Pro	
875–880	Hyp	
920–928	C─C stretching Pro	Collagen
932–941	Symmetric stretching	Collagen
932–938	C─C protein backbone	GAGs
937–941	C─O─Cα 1–4 glycosidic bond	
954–962	${\mathrm{PO}}_{4}^{3-}$, symmetric stretching	Phosphate hydroxyapatite (HA)
1004	Aromatic ring stretching phenylalanine (Phe)	Proteins
1039–1042	C─O─C stretching pyranose ring	GAGs
1047–1055	P─O─P symmetric stretching	CPPD
1060–1064	$O─{\mathrm{SO}}_{3}^{-}$ symmetric stretching	Sulphated GAGs, PGs
1070–1090	${\mathrm{CO}}_{3}^{2-}$, asymmetric stretching	Carbonate
1070–1073	Type-B carbonate	Carbonated hydroxyapatite
1080–1082	Amorphous carbonate	Amorphous carbonate
1090	${\mathrm{CaCO}}_{3}$	Calcium carbonate deposits
1125	Pyranose ring	GAGs
1230–1280	C─N stretching amide III	Collagen
1245	Random coil	Defective
1270	α-helix structure	Functional
1375–1380	${\mathrm{CH}}_{3}$ symmetric stretching	GAGs, PGs
1441–1460	${\mathrm{CH}}_{2}$ deformation/scissoring	Protein and lipids
1630–1690	C═O stretching amide I	Collagen and other protein
1645–1655	α-helix structure	
1660–1670	Random coil	
1665–1675	β-sheet structure	
1550–1600	N─H and C─N deformation amide II	Proteins

To characterize the spectral difference between the clusters, principal component loadings (PC loadings) were calculated and investigated in the same manner as the mean spectra [Figs. 2(b) and 2(c)]. The spectral feature of PC2 loadings exhibited a large and broad positive band around 850 to $940\;{\mathrm{cm}}^{-1}$ (unknown) and negative bands at $1061\;{\mathrm{cm}}^{-1}$ ($O─{\mathrm{SO}}_{3}^{-}$), $1125\;{\mathrm{cm}}^{-1}$ (pyranose ring), $1250\;{\mathrm{cm}}^{-1}$ (amide III), $1379\;{\mathrm{cm}}^{-1}$ (${\mathrm{CH}}_{3}$), and $1448\;{\mathrm{cm}}^{-1}$ (${\mathrm{CH}}_{2}$), which were due to PGs and GAGs [Fig. 2(b)]. The PC3 loadings had small positive peaks at $856\;{\mathrm{cm}}^{-1}$ (proline) and $936\;{\mathrm{cm}}^{-1}$ (C─C, C─O─C), which were assigned to collagen. These peaks indicate a countertendency compared to the PC2 loadings with regard to the Raman bands of PGs and GAGs [Fig. 2(c)]. These findings suggest that the use of PCA and HCA enables unsupervised classification of alteration patterns in the molecular composition of the cartilage matrix by patient. These patterns mainly consist of type II collagen, PGs, and GAGs.

**Fig. 3:**
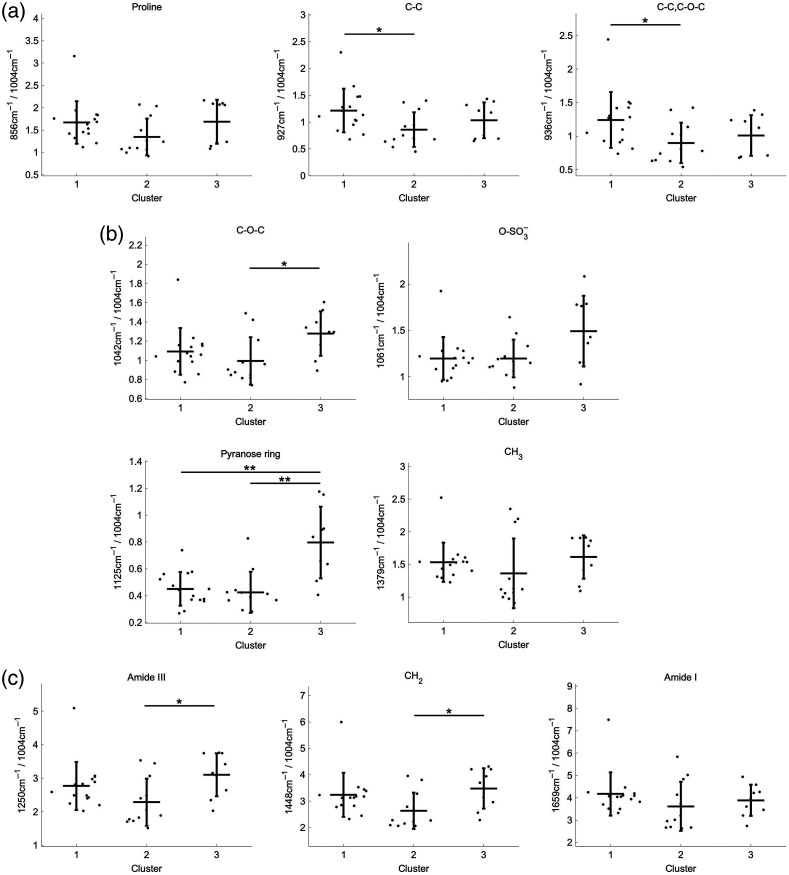
Quantitative results of the Raman ratios related to cartilage components. Values are mean ± SD. Symbols (•) represent individual samples. Significance is indicated as *p<0.05 and **p<0.01. (a)–(c) Quantitative results of the Raman ratios related to collagen, glycosaminoglycans, and proteins.

For a quantitative evaluation, we calculated and compared the relative intensities of 10 Raman bands using phenylalanine at $1004\;{\mathrm{cm}}^{-1}$ as a standard peak. Figure 3(a) represents the Raman peak intensity ratio at $856\;{\mathrm{cm}}^{-1}$ (proline), $927\;{\mathrm{cm}}^{-1}$ (C─C), and $936\;{\mathrm{cm}}^{-1}$ (C─C, C─O─C) of the three clusters. In cluster 1, the ratio of the Raman peaks associated with collagen tended to be higher compared to those of the other clusters. In cluster 2, the Raman intensity ratio of proline did not differ statistically, but those of the other peaks were statistically lower than those of cluster 1. In contrast, several Raman bands assigned to PGs and GAGs were larger in cluster 3 than the other clusters [Fig. 3(b)]. The peak at $1042\;{\mathrm{cm}}^{-1}$ (C─O─C) was significantly higher in cluster 3 than that for cluster 2. Additionally, the peak at $1125\;{\mathrm{cm}}^{-1}$ (pyranose ring) was significantly higher than those for the other two clusters. The intensity alteration of the peak at $1061\;{\mathrm{cm}}^{-1}$ ($O─{\mathrm{SO}}_{3}^{-}$) was insignificant, which was a similar tendency as the other peaks of PGs and GAGs. The peak at $1379\;{\mathrm{cm}}^{-1}$ (${\mathrm{CH}}_{3}$), which is associated with PGs and GAGs, did not show a significant difference. Figure 3(c) depicts the intensity ratio of typical Raman bands assigned to the cartilage matrix, amide III, ${\mathrm{CH}}_{2}$, and amide I. In cluster 3, amide III and ${\mathrm{CH}}_{2}$ were significantly higher compared to those of cluster 2. However, the clusters did not show a significant difference for amide I.

**Fig. 4:**
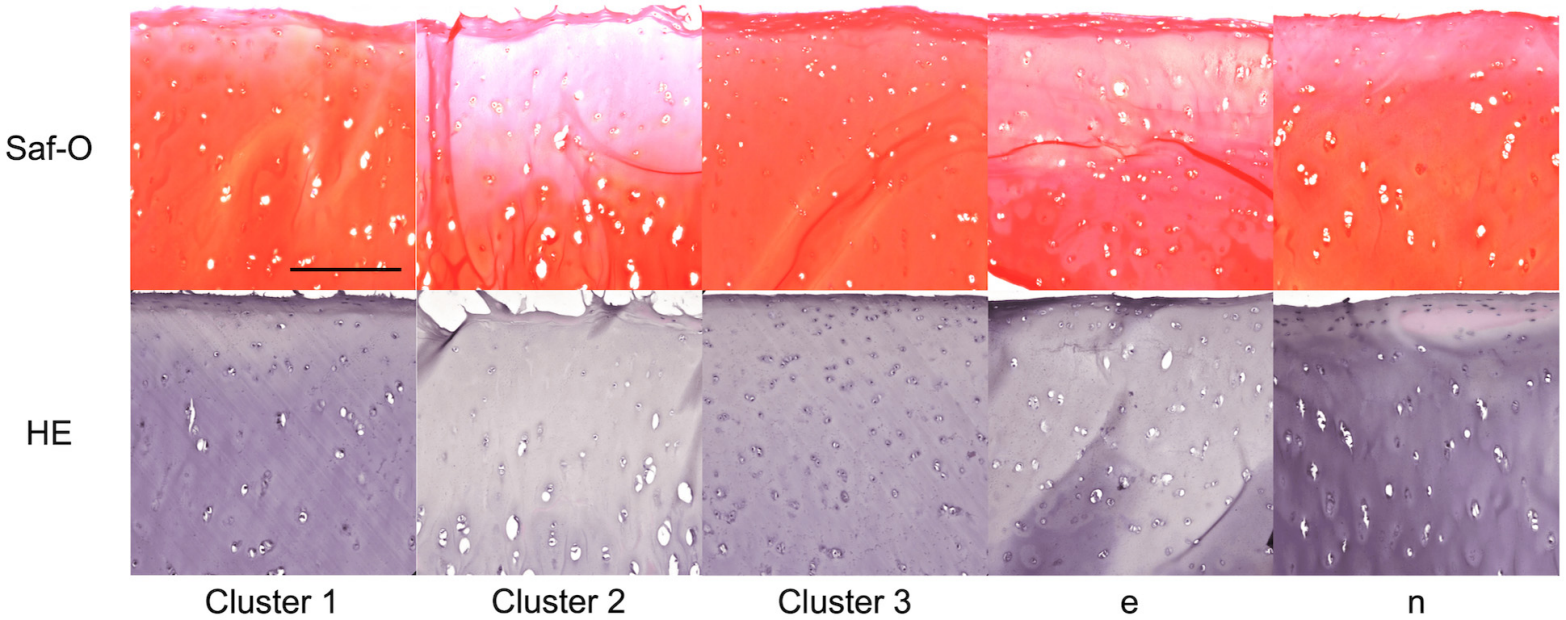
Histological image of osteoarthritic cartilage stained with Safranin–O and hematoxylin-eosin. From left to right: cluster 1, cluster 2, cluster 3, sample e, and sample n.

**Fig. 5:**
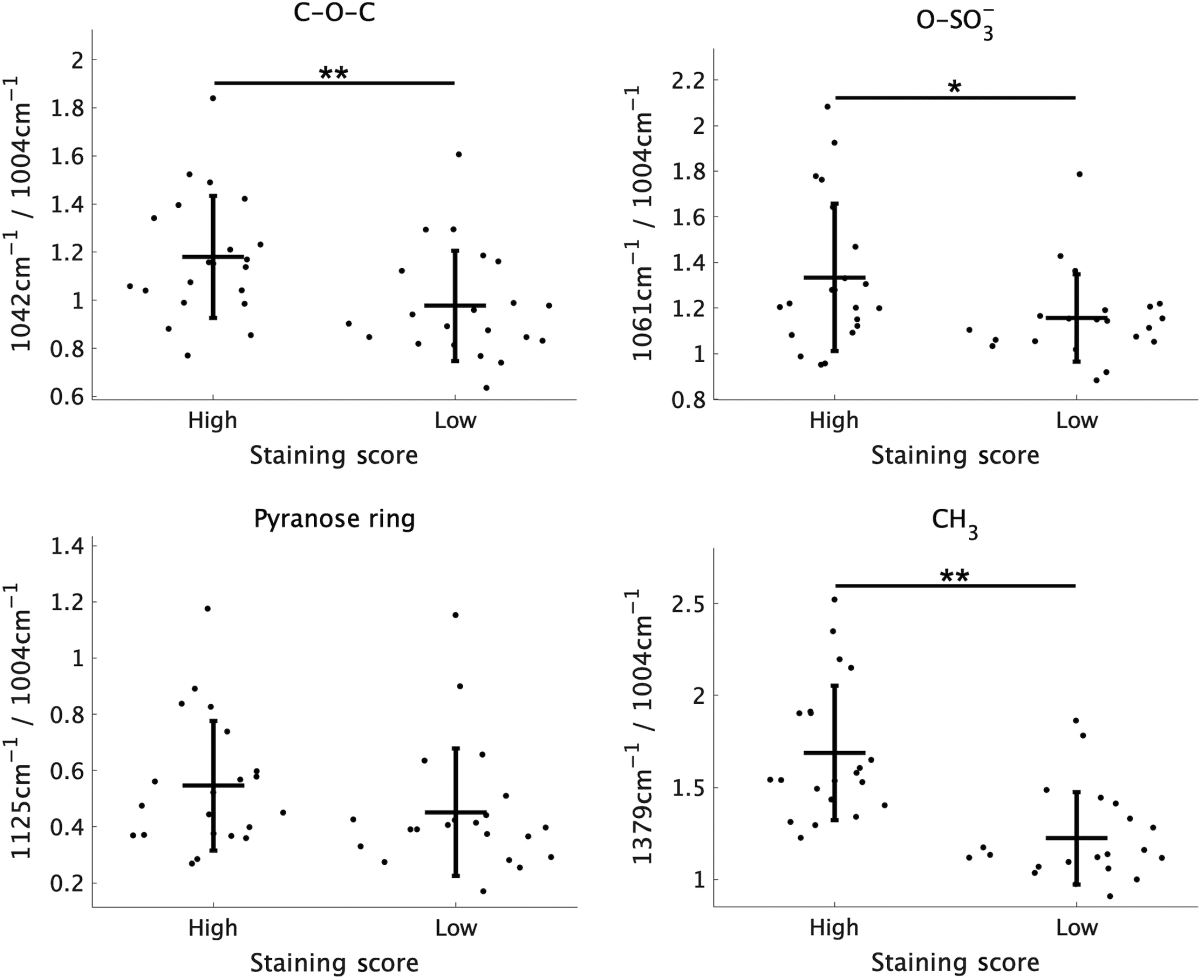
Correlations between the Raman relative intensity related to proteoglycans and the staining intensity of Safranin–O–staining (high, low). Significance is indicated as *p<0.05 and **p<0.01.

Histopathological analysis was performed on the serial section in which Raman spectral data was collected. Figure 4 shows representative histological images of the clusters and the other specimens (#e and #n) by Safranin–O–staining and HE–staining. Degenerative changes in the morphology and structure were detected in cluster 2. These changes were characterized by the cartilage surface roughness and weak staining. To assess the histological images quantitatively, the sections were classified into two groups (strong/weak). Figure 5 depicts the relationship between the Raman intensity ratio and the staining score of Safranin-O staining. The peaks of C─O─C, $O─{\mathrm{SO}}_{3}^{-}$, and ${\mathrm{CH}}_{3}$, and Saf–O staining score had positive correlations, but a significant correlation between the peak of pyranose ring and the staining score was not detected.

Table 2 presents the clinical characteristics of the patients whose Raman spectral data were clustered by PCA and HCA. Gender was a significant factor among the clusters (p<0.05) but not age. Patients in cluster 1 were all male. Conversely, patients in cluster 3 were all female. Cluster 2 included an equal number of male and female patients. Patients #e and #n, both were female, and the affected side was the dominant arm. Clusters 1 and 2 displayed a higher and lower tendency in the rate of dominant arm on the affected side, respectively. However, the difference was insignificant. Cluster 2 gave the highest scores in both K-L and ICRS grading compared with the other clusters and the patients. On the other hand, #e had the lowest OA score (grade II for K–L and ICRS) and was a case with a proximal humeral fracture. Most participants were diagnosed with rotator cuff tears in a shoulder joint, but cluster 2 included two patients (50%) with primary OA. An association between clustering and complications was not found, but most patients (80%) in cluster 1 had hypertension. In individual cases, OA of the knee and osteoporosis (#e) and dialysis (#n) were also included.

**Table 2:** Correlations between cluster samples and clinical findings. Values indicate the percentage of clinical findings included in the cluster.

	Cluster			p	Others	
	1 (n=5)	2 (n=4)	3 (n=3)		e	n
Age	76.6±7.26	76±4.64	73.3±3.3	0.813	74	71
Female	0 (0%)	2 (50%)	3 (100%)	0.027	T	T
Dominant arm	4 (80%)	1 (25%)	2 (66.6%)	0.267	T	T
KL classification (II:III:IV)	0 (0%):2 (40%):3 (60%)	0 (0%):0 (0%):4 (100%)	0 (0%):2 (66.6%):1 (33.3%)	0.192	II	II
ICRS classification (2:3:4)	0(0%):2 (40%):3 (60%)	0 (0%):0(0%):4 (100%)	0 (0%):1 (33.3%):2 (66.6%)	0.392	2	3
Diagnosis						
Osteoarthritis	0 (0%)	2 (50%)	0 (0%)	0.111	F	F
Rotator cuff tear	5 (100%)	2 (50%)	3 (100%)	0.111	F	T
Proximal humeral fracture	0 (0%)	0 (0%)	0 (0%)	N.A.	T	F
Complications						
Hypertension	4 (80%)	1 (25%)	1 (33.3%)	0.238	F	F
Diabetes mellitus	0 (0%)	1 (25%)	1 (33.3%)	0.438	F	F
Osteoarthritis of the knee	0 (0%)	0 (0%)	0 (0%)	N.A.	T	F
Dialysis	0 (0%)	0 (0%)	0 (0%)	N.A.	F	T
Osteoporosis	0 (0%)	0 (0%)	0 (0%)	N.A.	T	F

## 4 Discussion

This study aimed to reveal the correlation between typical clinical findings and alterations in the chemical composition of the cartilage matrix assessed by Raman spectroscopy in shoulder OA. Unsupervised machine learning methods successfully yielded a clinically meaningful classification between the shoulder OA patients. This approach not only has potential to confirm severity of cartilage defects but also to determine the origin of an individual’s OA by evaluating the cartilage quality. Often as knee and hip OA progresses, the hyaline cartilage layer is completely missing or replaced with fibrocartilage or ectopically formed bone. In contrast, our results indicate that for shoulder OA, even in the case of K–L grade IV, the hyaline cartilage layer remains on the humeral head. Preoperatively, similar to knee and hip OA, shoulder OA can be diagnosed according to radiographic scoring. However, the scoring does not necessarily follow that the severity of cartilage damage associated with the radiographical OA grade in the shoulder joint.^23^ To the best of our knowledge, this is the first report employing Raman spectroscopic analysis of humeral cartilage in shoulder OA.

To consider the abnormalities in the molecular composition of the cartilage matrix based on Raman spectroscopic analysis, previous studies have reported that several Raman bands, which are assigned to PG, GAG, and collagen contents, are reliable optical biomarkers for OA cartilage.^11^^,^^15^ Previous studies using K–L scoring^15^ and ICRS scoring^2^^,^^11^ found a negative correlation between the Raman peak intensities for PG and GAG contents and the degree of structural damage. This finding is partially consistent with our results for humeral cartilage. The Raman spectral data and Saf–O–staining for patients assigned to cluster 2, which have the severest case with K–L grade IV, show the lowest PG and GAG contents. Furthermore, the collagen content tends to decrease in cluster 2 compared to the other clusters. Interestingly, this is the only cluster that includes primary OA. All the other clusters involve tears of the rotator cuff, which may be the cause of secondary OA. Generally, degenerative changes observed in the humeral articular, which is a non–weight–bearing joint in secondary OA due to chronic instability or patients with a cuff tear arthropathy, may be less severe than those in weight-bearing joints such as hip and knee-joints.

This study has some limitations. First, the sample size of 14 patients is too small to explore the possible mechanism in primary OA. Although shoulder OA occurs less frequently than hip or knee OA, cohort studies with larger sample sizes are necessary to evaluate the potential influence of age, gender, comorbidities (e.g., diabetes mellitus, hypertensions, osteoporosis, OA in another joint), and environmental factors (e.g., smoking, alcohol, and dominant arm) on the onset of primary OA.^27^ As for epidemiological studies, hypertension has been associated with radiographic and symptomatic knee OA.^28^ Physioanatomically, articular cartilage has no blood vessels. Instead, oxygen and nutrients are supplied via perichondrium and the subchondral vascular system. A possible mechanism involved in the onset of primary OA is that systemic hypertension leads to subchondral bone perfusion abnormalities and ischaemia, impairing the maintenance function for chondrocyte and cartilage matrix.^28^ Another report suggested that hypertension also activates the renin–angiotensin, endothelin, and WNT-β-catenin signaling pathways, subsequently inducing cartilage degeneration. In this study, cluster 1 contained only male patients, and 80% had hypertension (Table 2). These patients showed lower contents of PGs and GAGs but inversely higher contents of collagen (Fig. 3). In contrast, cluster 3, which contained only female patients, a countertendency was found in the matrix composition. This fact suggests that the variety of compositional alterations in the cartilage matrix may depend on known risk factors, including age and gender related to the onset of primary OA. Although this study could not identify a significant association in aging and diabetes mellitus by Raman spectroscopic analysis, numerous reports have proposed that they are well-known risk factors in OA.^20^^–^^23^^,^^28^^–^^31^ These factors have been widely investigated in epidemiology, but the mechanism remains unclear. Regarding the individual cases (patient #e and n#), which were not included in any cluster, #e was the only case of arthroplasty due to a proximal fracture with osteoporosis and knee OA. Case #e exhibited unique behaviors in the Raman spectral data and the PCA score plot (Fig. 1). Case #n was a dialysis patient. Although kidney failure (CKD) generally affects bone and mineral metabolism, direct evidence for the association between CKD and OA has yet to be fully elucidated.

Another limitation of this study is that the excised specimens were fixed by formalin. Thus, its influence should be considered for pathological validation in clinical specimens. This is a preliminary ex vivo study toward the practical application of Raman arthroscopy for early treatment and improving prognosis of OA. As mentioned above, the advantage of Raman spectroscopic techniques is that they allow nondestructive measurements under arthroscopy.^32^ To date, Raman needle arthroscopy has been developed and reported.^33^ Kroupa et al.^34^ demonstrated Raman needle arthroscopy in both *ex vivo* and *in vivo* measurements for probing the cartilage surface of the knee joint. Our group has been developing a fiber-optic Raman spectroscopic system for endoscopic applications in animal and human optical biopsies.^35^^–^^37^ For *in vivo* assessments of the early stage of degenerative cartilage, the development of a Raman arthroscopy system for the shoulder joint is ongoing. Further studies with large size samples are being planned to comprehensively understand the mechanisms in onset and progression of OA.

## 5 Conclusion

We successfully demonstrated Raman spectroscopic analysis of humeral head, which was obtained from the patients who received shoulder arthroplasty. To our knowledge, this is the first study for shoulder OA designated to characterize degenerative cartilage in nonweight bearing joint by Raman spectral data and employing multivariate analyses. Compositional alterations in cartilage matrix of each patient’s cluster or each patient could link to known risk factors in primary and secondary OA. Our strategy advances an early diagnostic device and method based on Raman spectroscopy in combination of fiber–optic technique under arthroscopy toward minimally invasive screening system for early detection of OA and real-time monitoring system for treatment outcomes during OA therapies.
